# Ab Initio Investigation of the Adsorption of CO_2_ Molecules on Defect Sites of Graphene Surfaces: Role of Local Vacancy Structures

**DOI:** 10.3390/ma16030981

**Published:** 2023-01-20

**Authors:** Cui Wang, Ziming Wang, Shujie Zhang, Jianliang Zhang, Kejiang Li

**Affiliations:** 1 State Key Laboratory of Advanced Metallurgy, University of Science and Technology Beijing, Beijing 100083, China; 2 Department of Automotive Engineering, Hebei Vocational University of Technology and Engineering, Xingtai 054000, China; 3 Hebei Special Vehicle Modification Technology Innovation Center, Xingtai 054000, China; 4 School of Metallurgical and Ecological Engineering, University of Science and Technology Beijing, Beijing 100083, China

**Keywords:** CO_2_ adsorption, graphene defects, vacancy, density functional theory

## Abstract

An in-depth investigation into the adsorption of CO_2_ on graphene vacancies is essential for the understanding of their applications in various industries. Herein, we report an investigation of the effects of vacancy defects on CO_2_ gas adsorption behavior on graphene surfaces using the density functional theory. The results show that the formation of vacancies leads to various deformations of local carbon structures, resulting in different adsorption capabilities. Even though most carbon atoms studied can only trigger physisorption, there are also carbon sites that are energetically favored for chemisorption. The general order of the adsorption capabilities of the local carbon atoms is as follows: carbon atoms with dangling bonds > carbon atoms shared by five- and six-membered rings and a vacancy > carbon atoms shared by two six-membered rings and a vacancy. A stronger interaction in the adsorption process generally corresponds to more obvious changes in the partial density of states and a larger amount of transferred charge.

## 1. Introduction

The interactions between graphene-based carbons, i.e., graphite, coke, coal, etc., with gases containing oxygen, such as O_2_, NO, H_2_O and CO_2_ [1,2,3,4,5], have been the most important reactions in the chemical industry since the Industrial Revolution [6]. The thermodynamics and kinetics of reactions between oxygen-containing gases and various carbons have been significantly investigated [6,7,8,9]. These reactions are currently receiving great interest in materials-related applications, ranging from gas sensors [10,11,12], nanowindows [10] and fuel cells [13,14,15,16] to aircraft and space vehicles [5,17]. The sequestration of the greenhouse gas CO_2_ is currently one of the most pressing issues in environmental engineering because the increase in atmospheric CO_2_ concentration has been found to be the main cause of global warming [18]. This makes the study of the interaction of CO_2_ with various adsorption materials of great interest. With a both low cost and a high surface area, carbonaceous materials are becoming the most promising materials for capturing gas molecules [19,20]. Graphene, with many extraordinary properties, including an unparalleled large surface area and a high porosity, is currently receiving significant attention as a material that can be used to adsorb gas molecules from the atmosphere [21,22,23,24,25]. There has also been a lot of research on graphene defects, including the energetics and surface properties of graphene and graphene-like materials, using ab initio molecular dynamics (AIMD) [26,27]. The adsorption of CO_2_ on graphene surfaces has been experimentally and theoretically investigated in the past few years [9,22,23,28,29].

The interaction between CO_2_ molecules and graphene sheets is influenced by the surface characteristics, including the inherent electronic structure, the presence of defects [30,31], doping [12] and functionalized edge sites [32]. Perfect graphene sheets have a high chemical stability and unique physical properties due to the strong π-interaction of their hexagonal ring [33,34]. When a structural defect is formed in a graphene structure, it serves as a reactive site due to the locally increased reactivity of π electrons in the system [35]. A comprehensive and deep understanding of CO_2_ gas adsorption on defected graphene can be useful for the development of practical graphene-based gas sensors and for the understanding of CO_2_ reactions with graphene in other areas.

Previous studies have suggested that defects in graphene can enhance CO_2_ sensing properties; however, in these studies, only one type of defect was considered at a time [12,30]. Akilan et al. [12] recently investigated CO_2_ adsorption on four types of graphene defects (5-8-5, 55-77, 555-777 and 5555-6-7777) by removing one to two carbon atoms and rotating the carbon bonds, and the density functional theory (DFT) results with the Perdew–Burke–Ernzerhof (PBE) functional showed that only weak physical adsorption occurred, with an adsorption energy ranging within −0.085~−0.064 eV. Murugan et al. [30] studied the adsorption of CO_2_ on Stone–Thrower–Wales defects, and the physical adsorption energy was in the range of −0.051~−0.042 eV. The adsorption of CO_2_ on various graphene defects has been found to be physical due to the formation of various ring structures with 5, 6, 7, 8, 9 or a larger number of carbon atoms. The formation of ring structures in a defected graphene structure impedes the formation of dangling bonds, which have been found to be the true active sites for reactions [21,36]. Liu et al. [37] found that the physisorption energy of CO_2_ on monovacancy sites (~0.21 eV) is approximately 4 times as strong as that on a perfect graphene surface (~0.05 eV) and that the adsorption of CO_2_ on various defects varies significantly due to differences in the local electronic structures. Even though it is widely accepted that graphene sheets terminated with hydrogen can be used to simulate graphene in DFT calculations [38,39,40,41,42], studies have also shown that termination with different elements, such as fluorine and nitrogen, produces different results [12,30]. Graphene sheets without termination in a periodical boundary might produce a result closer to the actual situation [21]. In addition, in previous studies, several carbon atoms were fixed in the DFT geometry optimization process of defected graphene, which led to an artificially induced contraction of the lattice and the subsequent asymmetric chemisorption of CO_2_ [9,21,37]. Hence, a systematic study is needed to compare various defects on graphene and its influence on the CO_2_ adsorption process; such a study will provide a clear understanding of the applicability of graphene-based gas sensors and their reactions in various gasification processes.

Herein, we report an investigation on the effects of vacancies on CO_2_ gas adsorption behavior on graphene surfaces using the plane-wave electronic density functional theory. A more accurate picture of the mechanism of CO_2_ adsorption on defective graphene surfaces was obtained by investigating the effect of the local atomic structure and the electronic structure on the adsorption configuration and energy. It was found that the local states of carbon atoms in various vacancy structures influence their adsorption capabilities significantly. Even though most carbon atoms studied could only trigger physisorption, there were also carbon sites that were energetically favored for chemisorption. This paper provides scientific knowledge for the design of effective graphene and other gas sensors based on two-dimensional materials.

## 2. Computational Details and Models

Plane-wave-based DFT, as implemented in Quantum Espresso software [43], was adopted in the simulations. It was known that the advantage of the PBE [44] functional is that it describes the localized effects with strong bonds, while its disadvantage is that it accounts for long-range nonlocal effects with vdW forces, which have a long decay tail. Several functionals have been proposed to help better simulate weak interactions, and among them, the vdW-DF approach provides a generalized truly nonlocal functional independent of system geometry [45]. The vdW-DF2 functional [46] was used in this study. This functional is a new implementation of an earlier vdW-DF [47], and it replaces the revPBE semi-local exchange functional with PW86 and uses an improved large-N expansion asymptotic gradient correction for the long-range nonlocal component of the exchange-correlation energy. Vanderbilt ultrasoft pseudopotentials were adopted to describe the interactions between the valence electrons and the ionic core [48]. The kinetic energy cutoff for the wavefunction expansion was set at 65 Ry (1 Ry = 13.61 eV), while that for the charge density was set at 650 Ry. The convergence threshold for the self-consistent calculation was set at 1 × 10^−5^ Ry. All the graphene structures with various defects were firstly relaxed with variable cell sizes and then relaxed with the equilibrium cell size obtained from the adsorption calculations. All the relax calculations were conducted using conjugate gradient minimization until the magnitude of the residual force on each atom was less than 1 × 10^−3^ Ry/Bohr and the total energy residual was within 1 × 10^−3^ Ry. Brillouin zone integrations were performed using a Monkhorst-Pack grid with 8 × 8 × 1 k-points [49].

**Figure 1:**
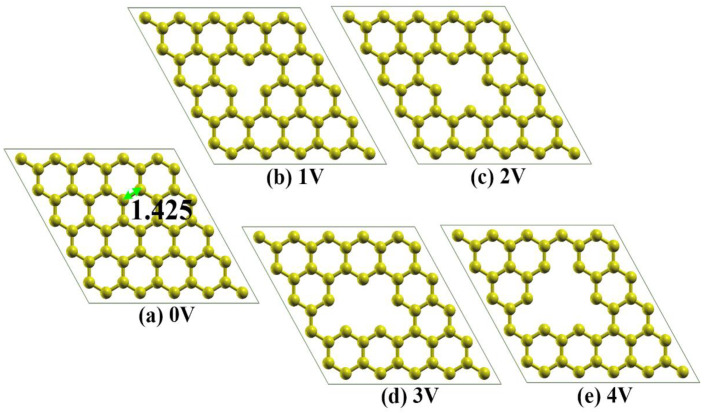
The basic structure of graphene for CO_2_ gas adsorption: (**a**) represents the pristine graphene (0V); (**b**–**e**) represents the carbon deficiency varying from monovacancy (1V), divacancy (2V) and trivacancy (3V) to tetravacancy (4V). The black numbers in the images represent the bond lengths.

All the basic graphene structures interacting with CO_2_ are shown in Figure 1. The modeled systems were all based on a supercell of 5 × 5 graphene containing 50 carbon atoms (Figure 1a). To simulate the effects of the defects, we removed carbon atoms to produce graphene with vacancies ranging from monovacancy (1V), divacancy (2V) and trivacancy (3V) to tetravacancy (4V), as shown in Figure 1b–e. All these defects have been predicted or observed in previous experiments [50,51,52,53,54]. The CO_2_ gas adsorption energy *E*a was calculated using the following equation:

*E*_a_ = *E*_sub+CO2_ − *E*_sub_ − *E*_CO2_, (1)

where *E*_sub+CO2_ and *E*_sub_ represent the energy of the relaxed pristine or the defective graphene substrate after and before CO_2_ gas adsorption, respectively. *E*_CO2_ represents the energy of a single CO_2_ gas molecule. It should be pointed out that an obvious structural deformation of the defective graphene and CO_2_ was observed in several cases after the CO_2_ adsorption process. Therefore, the deformation energy of the graphene substrate and that of CO_2_ after the adsorption were calculated using the following equations:

*E*_d_(sub) = *E*_sub_’ − *E*_sub_, (2)

*E*_d_(CO_2_) = *E*_CO2_’ − *E*_CO2_, (3)

where *E*_sub_’ and *E*_CO2_’ represent the energy of the graphene substrate and CO_2_ after the adsorption of CO_2_, respectively. This can be obtained by only calculating the energy of target atoms (graphene or CO_2_) while eliminating the other atoms after the adsorption process. Therefore, the interaction energy (*E*_i_) between the graphene substrate and CO_2_ after the adsorption process can be calculated using the following equation:

*E*_i_ = *E*_sub+CO2_ − *E*_sub_’ − *E*_CO2_’*,* (4)

The interaction energy (*E*_i_) can be used to calculate the strength of the connection between the graphene substrate and the adsorbed CO_2_. It should be mentioned that the final CO_2_ gas adsorption site for each type of defected structure was determined upon comparing the CO_2_ gas adsorption energies of all possible adsorption sites, which is discussed in the next section.

## 3. Results and Discussion

### 3.1. Structural Scrutiny of Defected Sheets and Determination of Adsorption Sites

**Figure 2:**
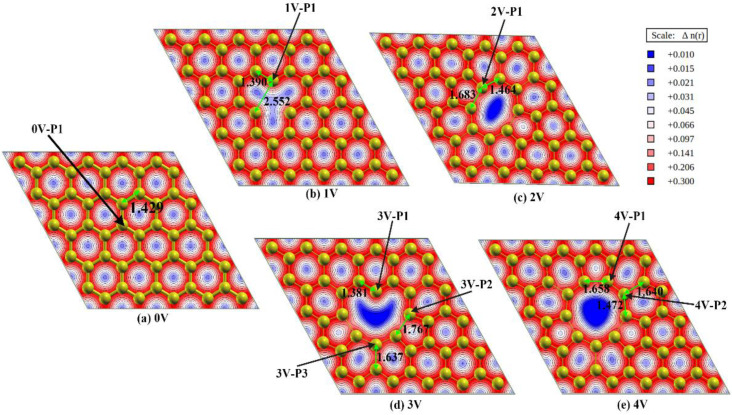
Optimized structures of graphene with various vacancies and their corresponding charge density distributions. All the possible adsorption sites are marked, and the arrangements of CO_2_ molecules on the adsorption sites are introduced in the Supplementary Materials. The black numbers in the images represent the bond lengths. (**a**–**e**) show the results of 0V–4V respectively.

The optimized structures of graphene with various vacancies and their corresponding charge density distributions are shown in Figure 2. It can be seen that there is no deformation of the pristine graphene, and the C-C bond length is almost unchanged (from an initial length of 1.425 Å to a relaxed length of 1.429 Å), while obvious local deformations to various extents can be observed for the carbon structures near the various vacancies. The elimination of the carbon atoms from the graphene network leads to the formation of three kinds of carbon atoms: (i) carbon atoms with a dangling bond and shared by only one six-membered ring (see 1V-P1 and 3V-P1 in Figure 2); (ii) carbon atoms that are shared by one five-membered ring and one six-membered ring (see 2V-P1, 3V-P2 and 4V-P1 in Figure 2); and (iii) carbon atoms that are shared by two six-membered rings (see 3V-P3 and 4V-P4 in Figure 2). Considering the equivalent positions, the seven positions mentioned above and marked in Figure 2 represent all the possible active sites for CO_2_ adsorption. The formation of a dangling bond leads to a decrease in the local bond length (from 1.429 Å to 1.390 Å in 1V-P1 and to 1.381 Å in 3V-P1), while the formation of a five-membered ring leads to an increase in the local bond length to various extents in different vacancies. The bond lengths in the five-membered rings in the 2V, 3V and 5V structures are 1.683 Å, 1.767 Å and 1.658 Å, respectively.

The adsorption routine is influenced by the arrangement of the CO_2_ molecules near the adsorption site. Recent studies [21,37,55] have found that chemical adsorption can only occur on active carbon sites, which should have dangling bonds, for instance, 1V-P1 and 3V-P1 in the present study. However, physical adsorption can occur on the top of any carbon atom [21,37], even though the adsorption strength varies in different local structures [22]. To include all the possible adsorption configurations and to also save computation resources, the possible physical adsorption configurations were computed for all the possible adsorption sites marked in Figure 2, while the possible chemical adsorption configurations were only considered for the active carbon sites with dangling bonds (1V-P1 and 3V-P1). In total, 14 initial configurations were considered, as shown in Tables S1–S4 in the Supplementary Materials, and each configuration was labeled with a unique name. Four kinds of initial configurations were included: (i) a configuration with the CO_2_ molecules parallel to the graphene substrate and with the carbon atoms of CO_2_ on the top of the target adsorption sites (see Table S1), labeled 2V-P1-ParalCO_2_-C; (ii) a configuration with the CO_2_ molecules perpendicular to the graphene substrate and with the oxygen atoms of CO_2_ on the top of the target adsorption sites (see Table S2), labeled 3V-P1-PerpenCO_2_-O; (iii) a configuration with the CO_2_ molecules parallel to the graphene substrate and with the carbon and oxygen atoms of CO_2_ on the top of the two target adsorption sites (see Table S3), labeled 3V-P1-ParalCO_2_-C-O; and (iv) a configuration with the CO_2_ molecules perpendicularly located in the vacancy and with the carbon atoms of CO_2_ close to the vacancy edge of the carbon atoms with dangling bonds (see Table S4), labeled 3V-P1-PerpenCO_2_-Edge. To characterize the interactions of the CO_2_ with the defects, the charge density after adsorption was calculated, while the partial density of states (PDOS) of the adsorption carbon atoms was compared with that of a reference carbon atom, which was far away from the vacancy.

### 3.2. Adsorption/Interaction/Deformation Energy and Charge Transfer

**Figure 3:**
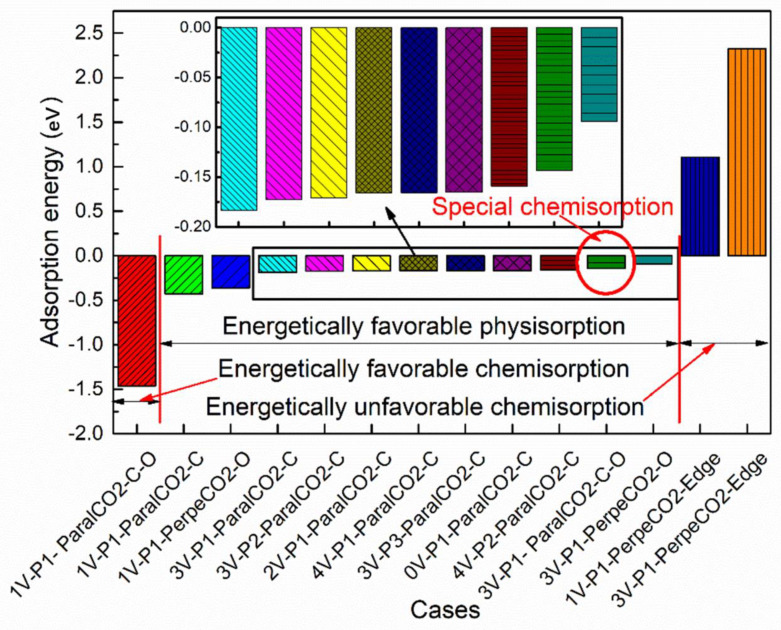
The adsorption energies of all the cases studied, with an increased order of adsorption energy. The inserted image is an amplification of the selected region showing the differences in physisorption energies.

**Figure 4:**
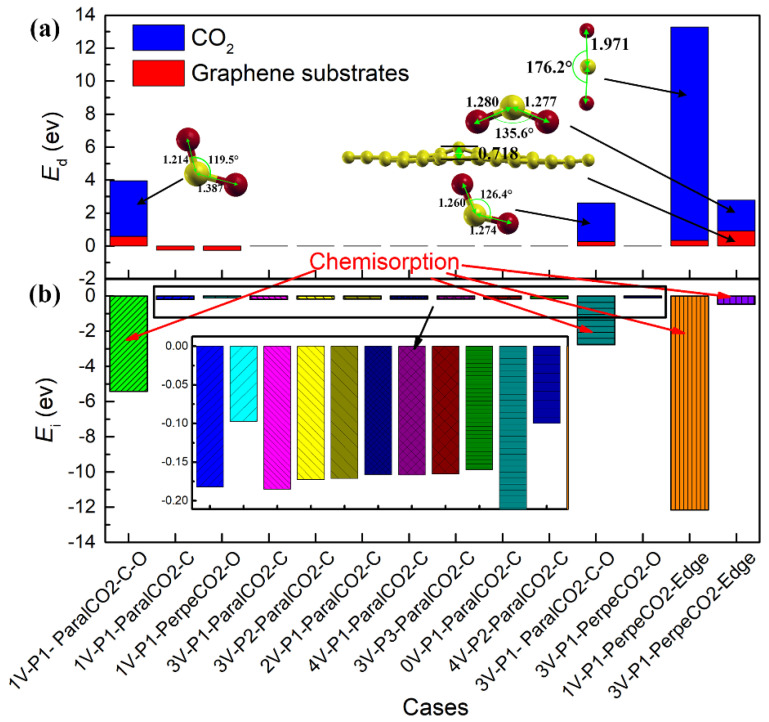
Deformation energy and interaction energy: (**a**) the deformation energy of graphene substrates and CO_2_ after adsorption process; (**b**) the interaction energy between the graphene substrates and adsorbed CO_2_.

The adsorption energies of all the cases studied are shown in Figure 3, with an increasing order of the adsorption energy. If the adsorption energy was chosen as the only criterion to distinguish physisorption from chemisorption, three kinds of adsorption would be observed: (i) energetically favorable physisorption for most cases, with an adsorption energy ranging from −0.428 eV to −0.093 eV; (ii) energetically favorable chemisorption for the case of 1V-P1-ParalCO_2_-C-O, with the most negative energy (−1.461 eV); and (iii) energetically unfavorable chemisorption for the cases of 1V-P1-PerpenCO_2_-Edge and 3V-P1-PerpenCO_2_-Edge, with a positive adsorption energy larger than 1 eV. However, the true difference between physisorption and chemisorption should be determined by the interaction bonds. Physisorption has the characteristic of weak van der Waals forces, while chemisorption has the characteristic of covalent bonding. To provide an overview of the bonding differences in these three kinds of adsorption, the deformation energy and interaction energy for each case were plotted, as shown in Figure 4a,b, respectively. With these new criteria, one special case (3V-P1-ParalCO_2_-C-O) was found. If only the adsorption energy was considered, this case would be considered representative of physisorption due to its low adsorption energy, but it should be considered representative of chemisorption because a strong interaction energy was observed and a covalent bond was formed (Figure 4a). This case is discussed in detail in Section 3.4. In Figure 4b, obvious deformations, especially of CO_2_ molecules, due to chemisorption can be observed regardless of whether the configurations are energetically favorable or unfavorable. The higher the dissociation energy, the lower the interaction energy. For 1V-P1-PerpenCO_2_-Edge, the dissociation of the oxygen atoms from the carbon atoms in the CO_2_ molecules required a very large deformation energy, making this configuration the most energetically unfavorable. The deformation energies of 1V-P1-ParalCO_2_-C-O and 3V-P1-PerpenCO_2_-Edge were very similar to each other, even though their adsorption energies varied greatly. The deformation energy and interaction energy of all the physisorption cases were very small and almost negligible. Since a larger interaction energy indicates a stronger bonding between the adopted deformed CO_2_ and graphene substrates, it is within expectation that chemisorption will lead to a much stronger bonding than physisorption.

**Figure 5:**
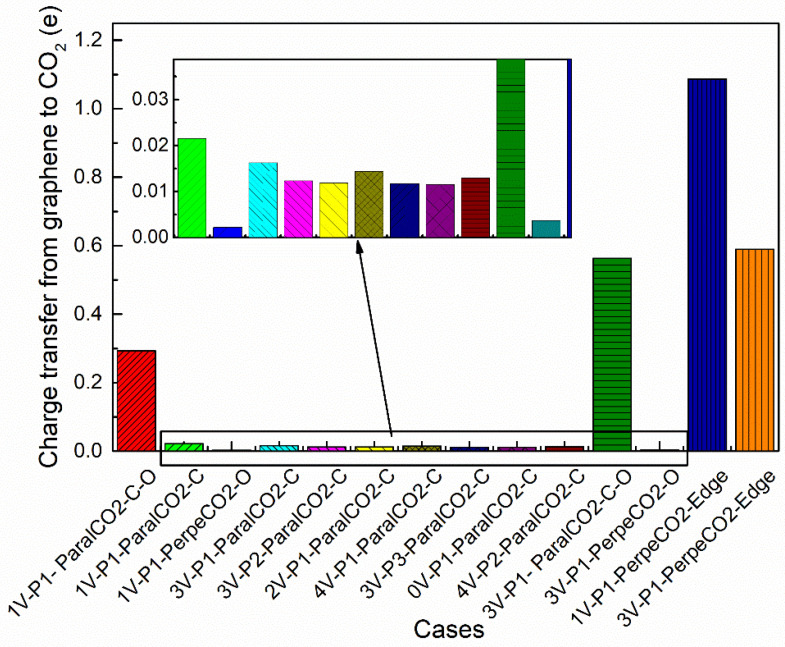
The Lowdin charge transfer from graphene substrates to CO_2_ in all cases.

The charge transfer was analyzed for all cases, and the results are shown in Figure 5. In the CO_2_ adsorption process, a clear charge transfer is observed from the graphene substrates to the CO_2_ molecules. This is understandable since the formation of defects leads to the less-saturated state of the adsorption carbon sites, especially for the sites of 1V-P1 and 3V-P1, where dangling bonds exist, and free outer shell electrons can move easily. In Figure 5, it can be seen that the charge transfer in physical adsorption (<0.025 e) is quite low compared with that in chemical adsorption (0.294~1.087 e), which is in good agreement with Noei’s study at the B3LYP level of theory [55]. The value of the charge transfer is closely related with the change in the charge density in the adsorption process, which is analyzed in detail in the next section.

### 3.3. Features of Physical Adsorption on Various Local Structures

#### 3.3.1. Physisorption on Pristine Graphene

**Figure 6:**
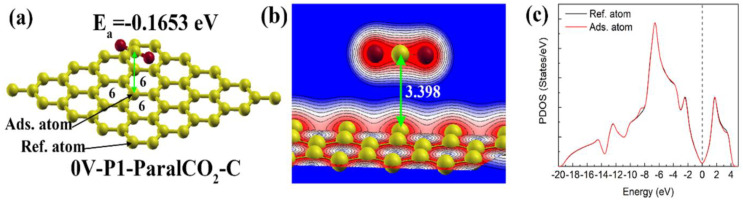
Physisorption configuration of CO_2_ on pristine (0V) graphene: (**a**) atomic structure; (**b**) charge distribution; (**c**) PDOS graph for the 2p orbital of reference carbon atom and adsorption carbon atom. The Fermi level is represented by the dashed vertical line.

The physical adsorption of gases is one of the most fundamental properties of graphene with or without defects [56]. In Figure 3, it can be seen that the physical adsorption energy varies from −0.429 to −0.0938 eV depending on the local defect structures and the adsorption configuration. The adsorption energy of CO_2_ on the pristine graphene (0V-P1-ParalCO_2_-C) obtained in the present study is −0.165 eV, which is in very good agreement with Takeuchi et al.’s results (−0.177 eV) [57] and the vdW-DF2 level of theory, and the vdW-DF2 adsorption energy is close to the experimentally reported adsorption energy (−0.271 eV). The adsorption energy obtained in this study is in good agreement with the adsorption energy reported in the literature, which verifies the simulation results. The distance between the CO_2_ and the adsorption site is 3.398 Å, and the atomic structure and the charge distribution are shown in Figure 6a,b, respectively. The charge distribution image indicates that there is no obvious electronic interaction between CO_2_. The partial density of state (Figure 6c) of the adsorption atom is almost the same as that of the reference atom, indicating that this physical interaction did not change the partial density of states.

#### 3.3.2. Physisorption on Carbon with Dangling Bond

**Figure 7:**
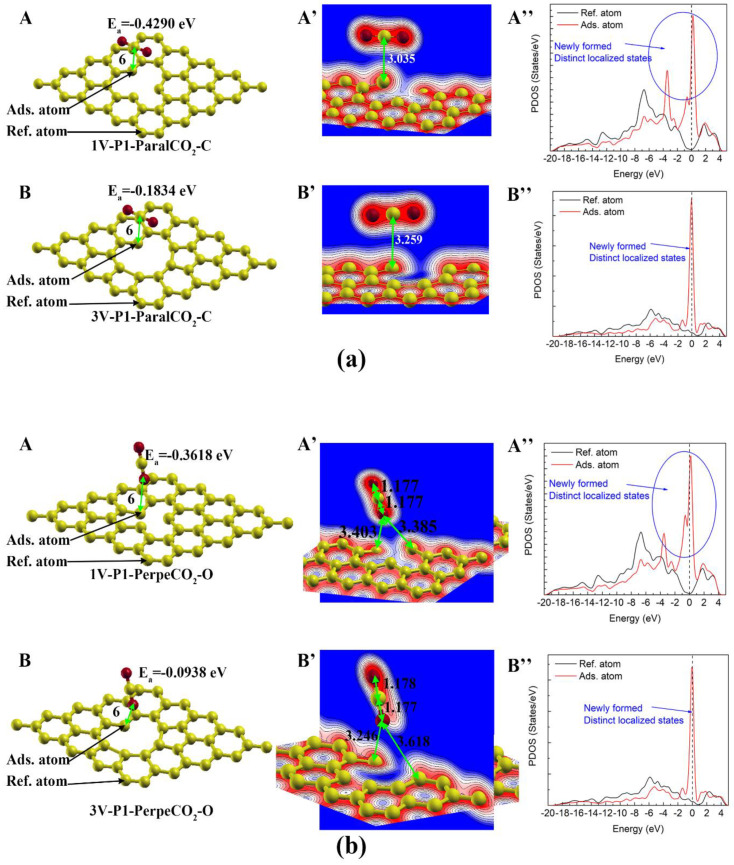
CO_2_ physisorption on carbon atoms with dangling bonds: (**a**) and (**b**) represent adsorption with CO_2_ molecules parallel to the graphene sheet and those perpendicular to the graphene sheet, respectively. (**A**,**A’**,**A’’**) and (**B**,**B’**,**B’’**) represent the atomic structure/charge distribution/PDOS of the adsorption configuration on monovacancy (1V) and trivacancy (3V) defects, respectively. The Fermi level is represented by the dashed vertical line.

Unsaturated carbon atoms are usually thought of as the most reactive sites on graphene sheets [22]. In the cases studied in the present study, there are only two positions (1V-P1 and 3V-P1) with unsaturated carbon atoms, as introduced in Section 3.1. The final adsorption configurations of the CO_2_ molecules parallel or perpendicular to the graphene sheets are shown in**Figure 7**. It can be seen that the adsorption capabilities of the 1V and 3V defects varies significantly, even though the adsorption carbon atoms in these two cases both have dangling bonds. The adsorption energy in the 1V-P1-ParalCO_2_-C case (−0.429 eV) is about two times less negative than that in the 3V-P1-ParalCO_2_-C case (−0.183 eV), with a much shorter adsorption distance between the center CO_2_ molecule and the adsorbed carbon atoms (see Figure 7a). In Figure 3, the 1V-P1-ParalCO_2_-C case represents the most negative adsorption energy among the physisorption cases, indicating that 1V-P1 has the strongest physisorption sites. In Figure 7a(A’’,B’’), it can be seen that the PDOS of the adsorbed carbon atoms changes obviously after the adsorption, with two newly formed sharp peaks, indicating the formation of distinct localized states.

By comparing the results in Figure 7a,b, it can be found that the adsorption with the carbon atoms of the CO_2_ molecules is more favorable than that with the oxygen atoms of the CO_2_ molecules since the adsorption energy in the first cases is more negative and has a shorter adsorption distance. Due to the strong adsorption capability of the 1V-P1 carbon, an obvious local deformation of the graphene network in the adsorption process can be observed. The carbon of CO_2_ seems to attract unsaturated carbon, with the adsorbed carbon moving upward (Figure 7a(A’)), while the oxygen of CO_2_ seems to repel unsaturated carbon, with the adsorbed carbon moving downward (Figure 7b(A’)). The adsorption with the oxygen of CO_2_ on the unsaturated carbon also leads to a change in the PDOS due the change in the local states (Figure 7b(A’’,B’’)).

#### 3.3.3. Physisorption on Carbon Shared by Five-Membered Rings, Six-Membered Rings and a Vacancy

**Figure 8:**
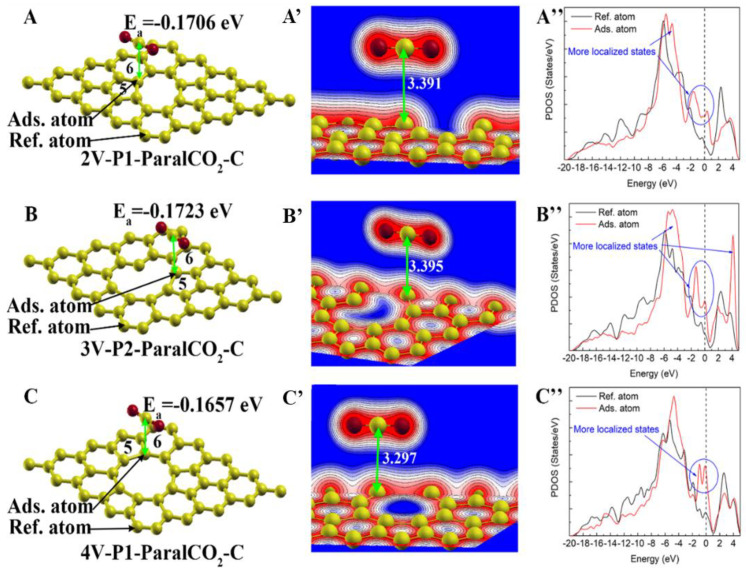
CO_2_ physisorption on carbon atoms shared by a 5-membered ring, a 6-membered ring and a vacancy: (**A**,**A’**,**A’’**), (**B**,**B’**,**B’’**) and (**C**,**C’**,**C’’**) represent the atomic structure/charge distribution/PDOS of adsorption configuration on divacancy (2V), trivacancy (3V) and tetravacancy (4V), respectively. The Fermi level is represented by the dashed vertical line.

Except for the formation of unsaturated carbon, the formation of a five-membered ring is another common phenomenon in various defected graphene structures [58]. Five-membered rings were observed in the 2V, 3V and 4V defects, and they usually neighbored six-membered rings. The carbon atoms shared by a five-membered ring, a six-membered ring and a vacancy seemed to have weak local bonding states compared with those of the pristine graphene carbon. Their atomic structure and final adsorption configurations are shown in Figure 8. Since the carbon of CO_2_ has a stronger adsorption potential than the oxygen of CO_2_, only the configurations with the adsorption of the carbon of CO_2_ were included in this experiment. It could be seen that the physisorption of CO_2_ on these adsorption sites was very weak, with their adsorption energy located within a very close range (−0.170~−0.166 eV), and their adsorption distances were very large (3.297~3.391 Å).

#### 3.3.4. Physisorption on Carbon Shared by Two Six-Membered Rings and a Vacancy

**Figure 9:**
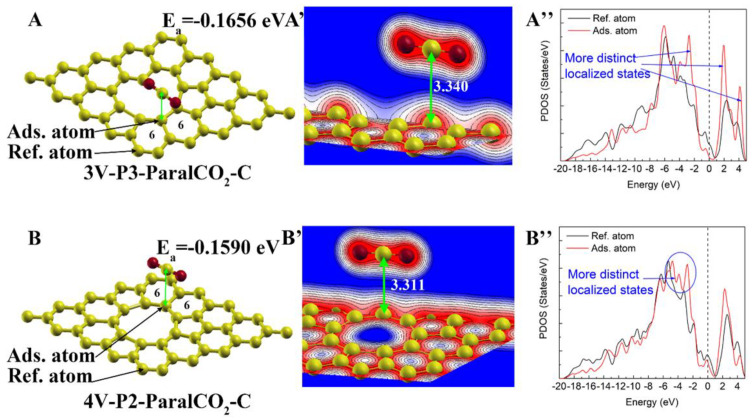
CO_2_ physisorption on carbon atoms shared by two 6-membered rings and a vacancy: (**A**,**A**’) and (**B**,**B’**) represent the atomic structure/charge distribution of adsorption on trivacancy (3V) and tetravacancy (4V) defects, respectively. A is the result of 3V-P3-ParaalCO_2_-C, and B is the result of 4V-P2-ParaalCO_2_-C.

The formation of a vacancy also leads to some edge carbon atoms that are shared by two six-membered rings and a vacancy. These kinds of carbon atoms should be more stable compared to the carbon atoms shared by a five-membered ring, a six-membered ring and a vacancy, since less structural deformation is required for these structures. The CO_2_ adsorption configurations on the carbon atoms shared by two six-membered rings and a vacancy are shown in Figure 9. The adsorption energy of 3V-P3-ParalCO_2_-C (−0.166 eV) is very close to that of pristine graphene (−0.165 eV), indicating that the local structure and properties of 3V-P3 did not change obviously. However, the adsorption energy of 4V-P2-ParalCO_2_-C is relatively larger than that of 3V-P3-ParalCO_2_-C and the pristine graphene, indicating that the adsorption capability of 4V-P2 is relatively weakened compared with that of the pristine graphene atoms.

### 3.4. Features of Chemical Adsorptions on Various Local Structures

#### 3.4.1. Energetically Favorable Chemisorption

**Figure 10:**
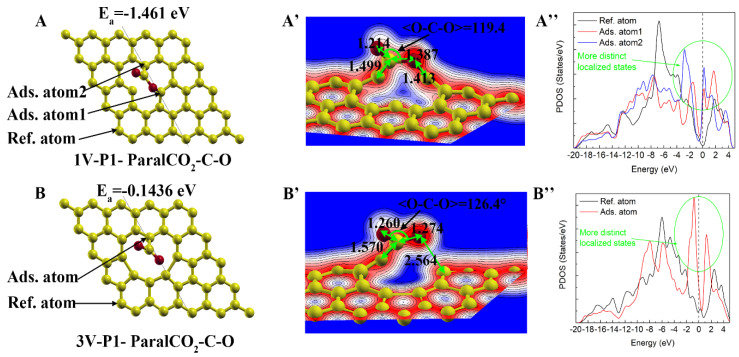
Energetically favorable chemisorption on carbon atoms with dangling bonds: (**A**,**A’**) and (**B**,**B’**) represent the atomic structure/charge distribution of adsorption on monovacancy (1V) and trivacancy (3V) defects, respectively. A is the result of 1V-P1-ParaalCO_2_-C-O, and B is the result of 4V-P2-ParaalCO_2_-C-O.

As introduced in Section 3.2, the case of 3V-P1-ParalCO_2_-C-O should be considered representative of chemisorption based on its highly negative interaction energy. Therefore, two energetically favorable chemisorption cases were observed in the present study, as shown in Figure 10. However, the adsorption energy of 3V-P1-ParalCO_2_-C-O (−0.144 eV) was much less negative compared with that of 1V-P1-ParalCO_2_-C-O (−1.461 eV), indicating that a monovacancy is more reactive than a trivacancy in triggering chemisorption. This difference is due to the difference in the local structure and the number of unsaturated carbon atoms. In the 1V structure, there were three unsaturated carbon atoms, and two of them formed strong bonds with the C and O of CO_2_ (see Figure 10A,A’). However, there was only one unsaturated carbon atom in the 3V structure, and only one strong chemical bond could be formed between the unsaturated carbon with the C of CO_2_, while only weak physical interactions could be observed between the O of CO_2_ and another vacancy edge carbon (similar to 3V-P3, which was shared by a five-membered ring, a six-membered ring and a vacancy). The PDOS also changed significantly after chemisorption. For both cases, more distinct localized states were observed around the Fermi energy level, while the main peak of the PDOS curve of the pristine graphene carbon located around −8~−5 eV decreased, as shown in Figure 10A’’,B’’.

#### 3.4.2. Energetically Unfavorable Chemisorption

**Figure 11:**
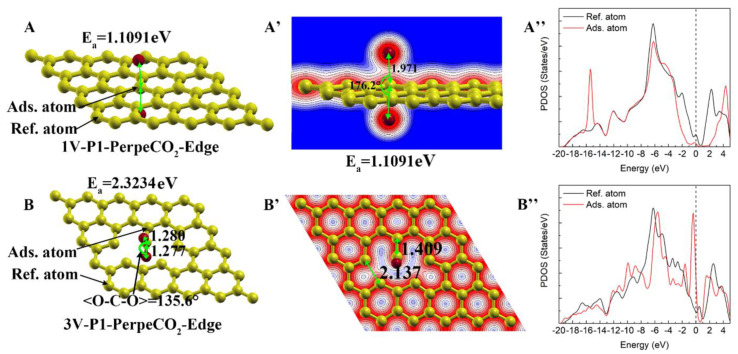
Energetically unfavorable chemisorption on carbon atoms with dangling bonds: (**A**,**A’**) and (**B**,**B’**) represent the atomic structure/charge distribution of adsorption on monovacancy (1V) and trivacancy (3V) defects, respectively. A is the result of 1V-P1-ParaalCO_2_-Edge, and B is the result of 3V-P1-ParaalCO_2_- Edge.

Two cases of energetically unfavorable chemisorption were observed, as shown in Figure 11. With a highly positive adsorption energy, these two cases cannot occur automatically, but it is possible if extra energy is provided. In the initial configurations of both cases, the CO_2_ molecules were perpendicularly located in the center of the vacancy (1V or 3V). For 1V-P1-PerpeCO_2_-Edge, it was found that the C=O bonds of CO_2_ were dissociated and that, subsequently, the C of CO_2_ formed a covalent bond with the surrounding three unsaturated carbon atoms, while only a weak interaction existed between the dissociated O and C (see Figure 11A,A’). The dissociation of the CO_2_ molecules required a lot of energy, resulting in a very high deformation energy of CO_2_ in this case, while the formation of the three covalent C-C bonds led to a very strong interaction energy between the adsorbed CO_2_ and the original 1V graphene structure (see Figure 4). For 3V-P1-PerpeCO_2_-Edge, a strong chemical bond was observed between the C of CO_2_ and the unsaturated carbon, while the angle of the <O-C-O> of the CO_2_ molecule was reduced to 135.6° (see Figure 11B,B’). Another feature of this adsorption was that the C-C bond of the five-membered ring that formed the vacancy was significantly weakened, with a bond length increase from 1.767 Å to 2.137 Å, and this also led an obvious deformation energy of the graphene (see Figure 4). For both cases, most of the PDOS curve of the adopted carbon overlapped with that of the pristine graphene carbon, while one newly formed sharp peak could be clearly observed. For the 1V-P1-PerpeCO_2_-Edge case, the newly formed peak was located at around −16 eV, while that of 3V-P1-PerpeCO_2_-Edge was located at around the Fermi energy level.

## 4. Conclusions

The CO_2_ adsorption on the possible adsorption sites of graphene defects with four kinds of vacancies was investigated systematically using the density functional theory. By analyzing the atomic and electronic structures, the adsorption and interaction energies, the charge transfer, and the PDOS, the following conclusions could be obtained:

The formation of vacancies led to various deformations of local carbon network structures by changing the local bond lengths and angles, resulting in different local environments or states of the carbon atoms. Carbon atoms with one dangling bond formed in the monovacancy and trivacancy structures, while those with five-membered rings easily formed in the divacancy, trivacancy and tetravacancy structures. The carbon atoms with one dangling bond and those shared by five-membered rings showed different adsorption capabilities from those shared by pristine six-membered rings.

Three CO_2_ adsorption types were observed among all the studied cases: (i) energetically favorable physisorption, with an adsorption energy range from −0.428 eV to −0.093 eV; (ii) energetically favorable chemisorption, with the most negative energy (−1.461 eV); and (iii) energetically unfavorable chemisorption, with a positive adsorption energy larger than 1 eV. The deformation energy and interaction energy for chemisorption were much larger than those for physisorption. The charge transfer in physical adsorption (<0.025 e) was quite low compared with that in chemical adsorption (0.294~1.087 e).

The adsorption of CO_2_ on the pristine graphene was quite weak, with a large adsorption distance (3.398 Å), and the partial density of state remained almost unchanged after adsorption. The carbon atoms with a dangling bond showed a stronger physisorption capability with a shorter adsorption distance (3.035~3.259 Å) and a significant change in the PDOS. The adsorption with the carbon atoms of the CO_2_ molecules was more favorable than that with the oxygen atoms of the CO_2_ molecules, which had a more negative adsorption energy. The CO_2_ adsorption capability on carbon atoms shared by a five-membered ring and a vacancy was much weaker than that on carbon atoms with dangling bonds, but it was stronger than that on carbon atoms shared by a five-membered ring and a vacancy.

The unsaturated carbon atoms of a monovacancy were more reactive than those of a trivacancy in triggering energetic favorable chemisorption. In the energetic favorable chemisorption process, more distinct localized states were observed around the Fermi energy level, while the main peak of the PDOS curve of the pristine graphene carbon located around −8~−5 eV decreased. For the energetic unfavorable chemisorption, most of the PDOS curve of the adopted carbon overlapped with that of the pristine graphene carbon, and one newly formed sharp peak was clearly observed.

In summary, this paper studied the adsorption of CO_2_ by different vacancies of two-dimensional graphene, and it carried out in-depth research on active adsorption sites, such as suspended carbon. At the same time, the use of the aperiodic boundary setting makes the simulation results more realistic than previous studies and provides a scientific basis for the design of efficient gas sensors, such as graphene-based sensors, using two-dimensional materials.

## Data Availability

Not applicable.

## Supplementary Materials

The following supporting information can be downloaded at https://www.mdpi.com/article/10.3390/ma16030981/s1, Table S1 Initial configurations for the adsorption with CO_2_ molecular parallel with graphene substrate and with carbon atom of CO_2_ on the top of target adsorption sites. The name of the configuration was shown below each image. Table S2 Initial configurations for the adsorption with CO2 molecular perpendicular to the graphene substrate and with oxygen atom of CO2 on the top of target adsorption sites. The name of the configuration was shown below each image. Table S3 Initial configurations for the adsorption with CO_2_ molecular parallel with graphene substrate and with carbon and oxygen atoms of CO_2_ on the top of two target adsorption sites. The name of the configuration was shown below each image. Table S4 Initial configurations for the adsorption with CO2 molecular in the vacancy and with carbon atom of CO_2_ close to the vacancy edge carbon atom with dangling bond. The name of the configuration was shown below each image.
