# Supplementary material for: Ab Initio Investigation of the Adsorption of CO2 Molecules on Defect Sites of Graphene Surfaces: Role of Local Vacancy Structures
Source: Materials (Basel). 2023 Jan 20;16(3):981. doi: 10.3390/ma16030981 (PMC9919361; doi:10.3390/ma16030981)
Supplement: Supplementary file 1 [file materials-16-00981-s001.zip › materials-2135949-supplementary.pdf]

Supporting Information

Ab-initio Investigation of the Adsorption of CO_2 Molecule on Defect Sites of Graphene Surface: Role of Local Vacancy Structures

Table of content

Table S1 Initial configurations for the adsorption with CO_2 molecular parallel with graphene substrate and with carbon atom of CO_2 on the top of target adsorption sites. The name of the configuration was shown below each image.

Table S2 Initial configurations for the adsorption with CO_2 molecular perpendicular to the graphene substrate and with oxygen atom of CO_2 on the top of target adsorption sites. The name of the configuration was shown below each image.

Table S3 Initial configurations for the adsorption with CO_2 molecular parallel with graphene substrate and with carbon and oxygen atoms of CO_2 on the top of two target adsorption sites. The name of the configuration was shown below each image.

Table S4 Initial configurations for the adsorption with CO_2 molecular in the vacancy and with carbon atom of CO_2 close to the vacancy edge carbon atom with dangling bond. The name of the configuration was shown below each image.

Supporting Information

Table S1 Initial configurations for the adsorption with CO_2 molecular parallel with graphene substrate and with carbon atom of CO_2 on the top of target adsorption sites. The name of the configuration was shown below each image.

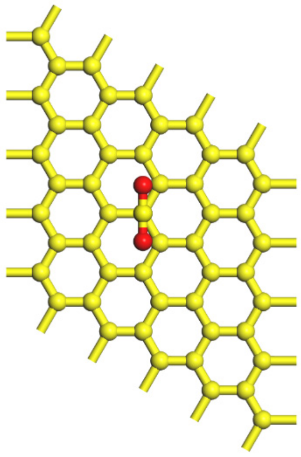<br>0V-P1-ParalCO_2-C	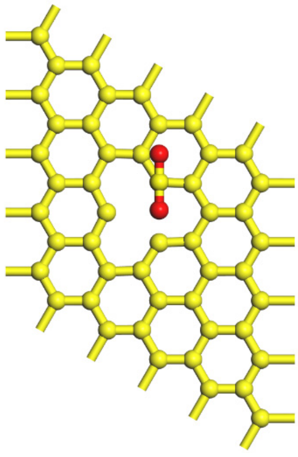<br>1V-P1- ParalCO_2-C	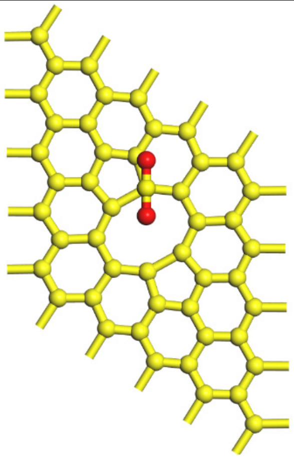<br>2V-P1-ParalCO_2-C
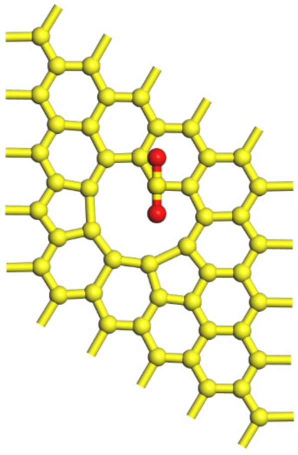<br>3V-P1-ParalCO_2-C	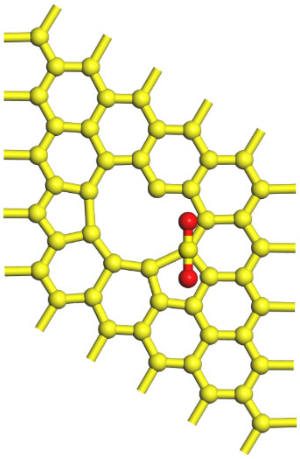<br>3V-P2- ParalCO_2-C	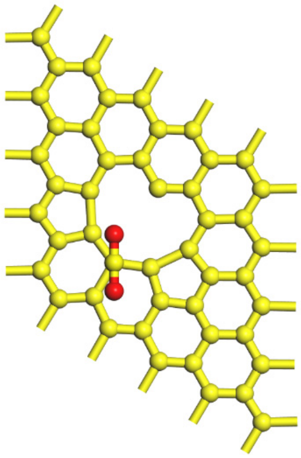<br>3V-P3- ParalCO_2-C
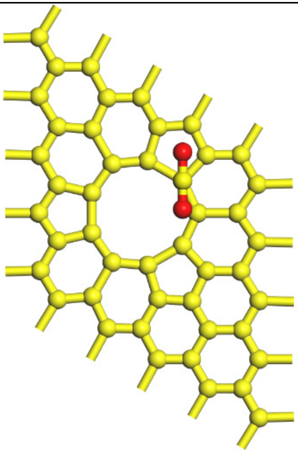<br>4V-P1- ParalCO_2-C	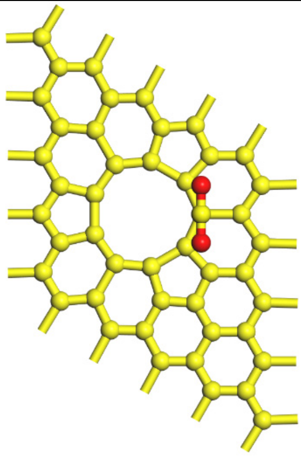<br>4V-P2- ParalCO_2-C	

Table S2 Initial configurations for the adsorption with CO_2 molecular perpendicular to the graphene substrate and with oxygen atom of CO_2 on the top of target adsorption sites. The name of the configuration was shown below each image.

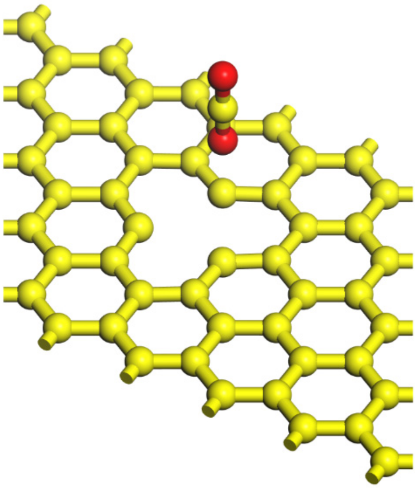<br>1V-P1-PerpenCO_2-O<br>1V-RP1	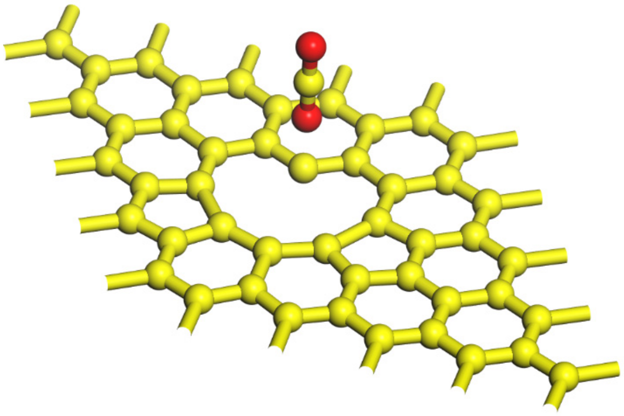<br>3V-P1-PerpenCO_2-O<br>3V-RP1

Supporting Information

Table S3 Initial configurations for the adsorption with CO_2 molecular parallel with graphene substrate and with carbon and oxygen atoms of CO_2 on the top of two target adsorption sites. The name of the configuration was shown below each image.

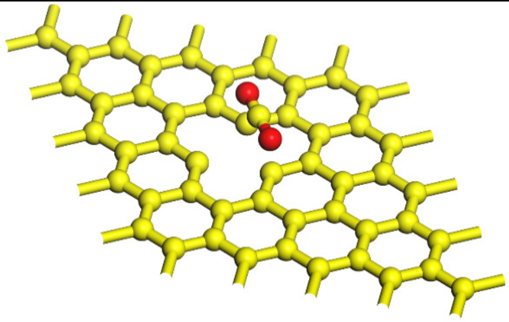<br>1V-P1- ParalCO_2-C-O<br>1V-RP2	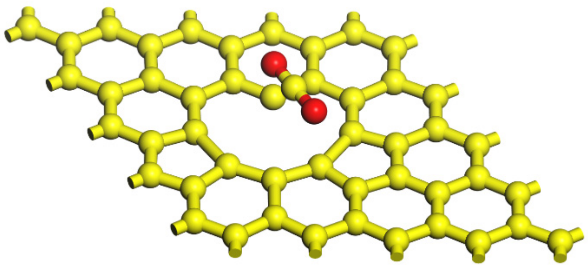<br>3V-P1- ParalCO_2-C-O<br>3V-RP2

Table S4 Initial configurations for the adsorption with CO_2 molecular in the vacancy and with carbon atom of CO_2 close to the vacancy edge carbon atom with dangling bond. The name of the configuration was shown below each image.

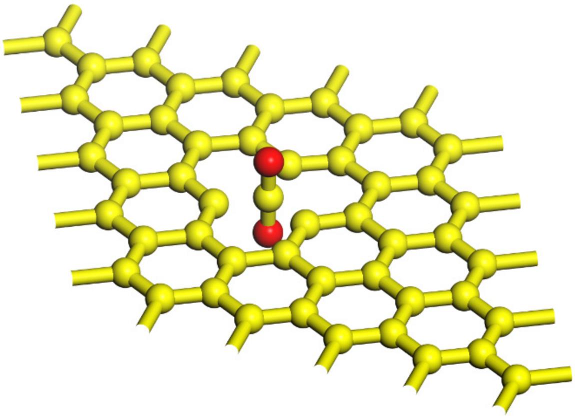<br>1V-P1-PerpenCO_2-Edge<br>1V-RP3	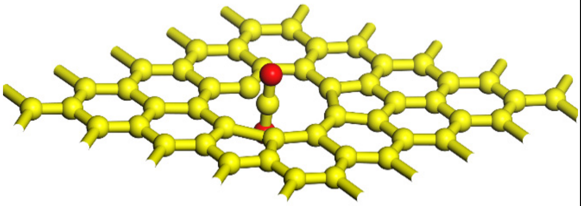<br>3V-P1-PerpenCO_2-Edge<br>3V-RP3
